# Risk Factors, Comorbidities, and Prevention of Cardiovascular Diseases: Don’t Forget the Primary Cause!

**DOI:** 10.3390/jcm13226652

**Published:** 2024-11-06

**Authors:** François Roubille

**Affiliations:** PhyMedExp, Cardiology Department, University of Montpellier, INSERM U1046, CNRS UMR, 9214, INI-CRT, 34090 Montpellier, France; f-roubille@chu-montpellier.fr

Cardiovascular diseases (CVDs) remain a leading cause of mortality worldwide. Despite innovative treatments, both pharmacological and interventional, CVDs continue to progress. This is largely due to the increased incidence of diseases such as diabetes and other well-established cardiovascular risk factors, as well as cumulative comorbidities and emerging risk factors.

For cardiologists, efficient therapies are often provided based on underlying causes and pathophysiological pathways. However, this is not always the case, as some mechanisms might remain hidden. Interestingly, until the recent development of drugs such as gliflozins, the cornerstone of managing heart failure (HF) with preserved left ventricular ejection fraction was primarily the control of comorbidities, cardiovascular risk factors, or concomitant disorders. In spite of these recent advances, we must not forget the crucial importance of a holistic approach to complex diseases such as HF, beyond the use of effective drugs. Clinicians dealing with CVD should be aware of new avenues in emerging CV factors, especially as they manifest sustained pathophysiological pathways that have likely been underconsidered until now.

Indeed, addressing comorbidities is not only a holistic approach and a reliable basis of medical practice but also involves considering new potential pathways that could offer more efficient management of various CVDs. Specific strategies could be appealing for particular populations subject to unique risk factors, including professional ones such as military personnel.

Classical risk factors are better delineated here, such as lipids and the common pathways between inflammatory processes and lipids, represented by lipoprotein A, or prognostic scores based on well-established and emerging factors. This can also help to better understand the impact of well-known factors (for instance, pericardial fat) and their specific pathways in order to propose tailored therapies.

Paradoxically, some classical risk factors may conceal specificities. This is the case for women, who are at high risk for peripheral artery disease [1], although cardiovascular risk in women is often largely underconsidered. A review is dedicated to this crucial topic.

In this special issue, some emerging risk factors are better depicted and their impact better understood, including psychiatric disorders and depression, sleep disorders and new adapted treatments, and low-grade inflammation in conditions such as osteoarthritis or vasculitis.

A holistic approach involves considering drug interactions, polypharmacy, and side effects in vulnerable populations. Similarly, interrelationships, such as those between metabolic syndromes and depression, could reinforce the deleterious effects of each other.

Such strategies could be tailored for both secondary and primary prevention. The potential of vaccines in these vulnerable populations deserves to be better known by clinicians, as they could offer easy and powerful tools at a nationwide level. From this initial statement [2], our group was able to build dedicated programs to overcome this barrier (NCT06360315), highlighting that reviews or Special Issues can be the first step towards the awareness necessary for further developments.

## References

[1] Mazzolai L., Teixido-Tura G., Lanzi S., Boc V., Bossone E., Brodmann M., Bura-Rivière A., De Backer J., Deglise S., Della Corte A. (2024). 2024 ESC Guidelines for the management of peripheral arterial and aortic diseases. Eur. Heart J..

[2] Girerd N., Chapet N., Roubille C., Roncalli J., Salvat M., Mouquet F., Lamblin N., Gueffet J.P., Damy T., Galinier M. (2021). Vaccination for Respiratory Infections in Patients with Heart Failure. J. Clin. Med..

